# Universal Definition of Loss to Follow-Up in HIV Treatment Programs: A Statistical Analysis of 111 Facilities in Africa, Asia, and Latin America

**DOI:** 10.1371/journal.pmed.1001111

**Published:** 2011-10-25

**Authors:** Benjamin H. Chi, Constantin T. Yiannoutsos, Andrew O. Westfall, Jamie E. Newman, Jialun Zhou, Carina Cesar, Martin W. G. Brinkhof, Albert Mwango, Eric Balestre, Gabriela Carriquiry, Thira Sirisanthana, Henri Mukumbi, Jeffrey N. Martin, Anna Grimsrud, Melanie Bacon, Rodolphe Thiebaut, on behalf of the International Epidemiologic Databases to Evaluate AIDS Collaboration

**Affiliations:** 1 University of Alabama at Birmingham, Birmingham, Alabama, United States of America; 2 Centre for Infectious Disease Research in Zambia, Lusaka, Zambia; 3 Indiana University, Indianapolis, Indiana, United States of America; 4 RTI International, Research Triangle Park, North Carolina, United States of America; 5 The Kirby Institute, University of New South Wales, Sydney, New South Wales, Australia; 6 Fundación Huésped, Buenos Aires, Argentina; 7 University of Bern, Bern, Switzerland; 8 Zambian Ministry of Health, Lusaka, Zambia; 9 INSERM U897, Bordeaux Segalen University ISPED, Bordeaux, France; 10 Instituto de Medicina Tropical Alexander von Humboldt, Universidad Peruana Cayetano Heredia, Lima, Peru; 11 Chiang Mai University, Chiang Mai, Thailand; 12 Amo-Congo, Kinshasa, Democratic Republic of the Congo; 13 University of California at San Francisco, San Francisco, California, United States of America; 14 University of Cape Town, Cape Town, South Africa; 15 National Institutes of Health, Bethesda, Maryland, United States of America; Duke University Medical Center, United States of America

## Abstract

**Background:**

Although patient attrition is recognized as a threat to the long-term success of antiretroviral therapy programs worldwide, there is no universal definition for classifying patients as lost to follow-up (LTFU). We analyzed data from health facilities across Africa, Asia, and Latin America to empirically determine a standard LTFU definition.

**Methods and Findings:**

At a set “status classification” date, patients were categorized as either “active” or “LTFU” according to different intervals from time of last clinic encounter. For each threshold, we looked forward 365 d to assess the performance and accuracy of this initial classification. The best-performing definition for LTFU had the lowest proportion of patients misclassified as active or LTFU. Observational data from 111 health facilities—representing 180,718 patients from 19 countries—were included in this study. In the primary analysis, for which data from all facilities were pooled, an interval of 180 d (95% confidence interval [CI]: 173–181 d) since last patient encounter resulted in the fewest misclassifications (7.7%, 95% CI: 7.6%–7.8%). A secondary analysis that gave equal weight to cohorts and to regions generated a similar result (175 d); however, an alternate approach that used inverse weighting for cohorts based on variance and equal weighting for regions produced a slightly lower summary measure (150 d). When examined at the facility level, the best-performing definition varied from 58 to 383 d (mean = 150 d), but when a standard definition of 180 d was applied to each facility, only slight increases in misclassification (mean = 1.2%, 95% CI: 1.0%–1.5%) were observed. Using this definition, the proportion of patients classified as LTFU by facility ranged from 3.1% to 45.1% (mean = 19.9%, 95% CI: 19.1%–21.7%).

**Conclusions:**

Based on this evaluation, we recommend the adoption of ≥180 d since the last clinic visit as a standard LTFU definition. Such standardization is an important step to understanding the reasons that underlie patient attrition and establishing more reliable and comparable program evaluation worldwide.

*Please see later in the article for the Editors' Summary*

## Abstract 2

Based on a statistical analysis of 111 facilities in Africa, Asia, and Latin America, Benjamin Chi and colleagues develop a standard loss-to-follow-up (LTFU) definition that can be used by HIV antiretroviral programs worldwide.

## Editors' Summary

**Background:**

Since 1981, AIDS has killed more than 25 million people, and about 33 million people (mostly in low- and middle-income countries) are now infected with HIV, the virus that causes AIDS. Because HIV destroys immune system cells, HIV-positive individuals are very susceptible to other infections, and, early in the AIDS epidemic, most HIV-infected people died within ten years of contracting the virus. Then, in 1996, antiretroviral therapy (ART)—a cocktail of drugs that keeps HIV in check—became available. For people living in developed countries, HIV infection became a chronic condition. However, for people living in developing countries, ART was prohibitively expensive, and HIV/AIDS remained a fatal illness. In 2003, this situation was declared a global emergency, and governments, international agencies, and funding bodies began to implement plans to increase ART coverage in resource-limited countries. By the end of 2009, more than a third of people living in these countries who needed ART were receiving it.

**Why Was This Study Done?:**

Because ART does not cure HIV infection, patients have to take antiretroviral drugs regularly for the rest of their lives. But in some ART programs, more than a third of patients are lost to follow-up (LTFU), that is, they stop coming for treatment, within three years of starting treatment. Patient attrition threatens the success of ART programs, but to understand why it occurs, a standardized method for classifying patients as LTFU is essential. Classification of patients as LTFU relies on an interval-based definition of LTFU. That is, a patient who fails to attend a clinic within a specified interval after a previous visit is classified as LTFU. If this interval is too short, although many patients will be accurately identified as LTFU, there will be a high false-positive rate—some patients classified as LTFU will actually return to the clinic later. Conversely, if the interval is too long, some patients who are truly LTFU will be misclassified as active (a false-negative classification). In this study, the researchers analyzed data from health facilities across Africa, Asia, and Latin America to determine a standard definition for LTFU that minimizes patient misclassification.

**What Did the Researchers Do and Find?:**

Using data collected from 111 health facilities by the International Epidemiologic Databases to Evaluate AIDS (IeDEA) Collaboration, the researchers categorized patients receiving ART at each facility at a “status classification” date (12 months before the facility's last data export to IeDEA) as active or LTFU using a range of intervals (thresholds) since their last clinic visit. For example, for a test interval of 200 days, patients who had not revisited the clinic within 200 days of their previous visit at the status classification date were classified as LTFU; patients who had revisited the clinic were classified as active. The researchers then looked forward 365 days from the status classification date to assess the performance and accuracy of these classifications. So, a “LTFU” patient who visited the clinic anytime during the year after the status classification date represented a false-positive classification, and an “active” patient who did not return within the ensuing year represented a false-negative classification. When data from all the facilities were pooled, a threshold of 180 days produced the fewest misclassifications. At the facility level, the best-performing threshold for patient classification ranged from 58 to 383 days (with an average of 150 days), but application of a 180-day threshold to individual facilities only slightly increased misclassifications. Finally, using the 180-day threshold, average LTFU at individual facilities was 19.9%.

**What Do These Findings Mean?:**

Based on these findings, the researchers recommend that the standard definition for LTFU should be when it has been 180 days or more since the patient's last clinic visit. Given the wide range of best-performing definitions among facilities, however, they recognize that local, national, or regional definitions of LTFU may be more appropriate in certain contexts. Adoption of a standard definition for LTFU, the researchers note, should facilitate harmonization of monitoring and evaluation of ART programs across the world and should help to identify “best practices” associated with low LTFU rates. Importantly, it should also provide the necessary framework for research designed to improve patient retention in ART programs, thereby helping to maximize and sustain the health gains from HIV treatment programs.

**Additional Information:**

Please access these websites via the online version of this summary at http://dx.doi.org/10.1371/journal.pmed.1001111.

- Information is available from the US National Institute of Allergy and Infectious Diseases on HIV infection and AIDS
- NAM/aidsmap provides basic information about HIV/AIDS and summaries of recent research findings on HIV care and treatment
- Information is available from Avert, an international AIDS charity on many aspects of HIV/AIDS, including information on HIV/AIDS treatment and care and on universal access to AIDS treatment (in English and Spanish)
- The World Health Organization provides information about universal access to AIDS treatment (in several languages)
- Information about the IeDEA Collaboration available
- Patient stories about living with HIV/AIDS are available through Avert and through the charity website Healthtalkonline

## Introduction

Unprecedented gains have been made in the expansion of services for antiretroviral therapy (ART) in resource-constrained settings. The United Nations Joint Programme for HIV/AIDS (UNAIDS) now estimates that more than 5 million HIV-infected adults and children have initiated HIV treatment worldwide, a 13-fold increase since 2003 [1],[2]. Patient attrition and losses to follow-up, however, have emerged as legitimate threats to the long-term success of these programs. A systematic review of sub-Saharan African cohorts reported lost to follow-up (LTFU) rates as high as 35% in the 3 y following ART initiation [3],[4], a finding supported by other regional reports [5]–[7].

Although commonly described in the context of ART programs, the accurate categorization of a patient as either active or LTFU presents unique challenges. An inherent risk to interval-based definitions for LTFU is misclassification, since a patient who is late for a clinic appointment may elect to return even after the window has elapsed. An interval that is close to the visit date, for example, may be highly sensitive (i.e., a high proportion of patients are accurately identified as LTFU), but specificity will be low. Conversely, an interval that is long will be highly specific (i.e., a high proportion of patients are accurately classified as active), but sensitivity may be limited.

We developed a methodology to empirically determine the optimal operational definition for LTFU [8]. Applied to a cohort of 33,704 ART patients in Lusaka, Zambia [9],[10], we found that a threshold of ≥56 d since last missed visit led to the fewest misclassifications of a patient's status as active or LTFU (5.1%, 95% confidence interval [CI]: 4.8%–5.3%). The primary limitation of our analysis, however, was its external validity. Because participating facilities in Lusaka shared many characteristics (e.g., urban locale, free care, active contact tracing program), their definition for LTFU might not be appropriate for other populations. In this report, we apply this methodology to 111 health facilities across three continents and generate an evidence-based LTFU definition for ART program evaluation worldwide.

## Methods

To empirically determine the best-performing definition for LTFU among adults (i.e., >16 y at ART initiation) on ART, we analyzed data from six of the seven regions of the International Epidemiologic Databases to Evaluate AIDS (IeDEA) Collaboration: Central Africa, Eastern Africa, Southern Africa, Western Africa, Asia/Pacific, and Latin America/Caribbean. Across these regions, HIV programs from 41 countries contribute unlinked and anonymous individual-level data to the IeDEA initiative [11]. Use of these observational data has been approved by ethics committees and/or institutional review boards in host countries, as well as through those of international partners. Data for each of the regions are updated at least annually and managed by RTI International (Central Africa), Indiana University (Eastern Africa), University of Bern and University of Cape Town (Southern Africa), University of Bordeaux (Western Africa), University of New South Wales (Asia/Pacific), and Vanderbilt University (Latin America/Caribbean).

Because many programs do not routinely collect information regarding the date of next clinic visit, a “days late” definition for LTFU—as used in our Lusaka cohort [8]—may be challenging to implement globally. For that reason, we sought an optimal LTFU definition based on the number of days since last clinic encounter. Our unit of interest was at the level of the health care facility, also described as “health centers” in this report. To ensure adequate patient follow-up time, we included only health centers with a minimum of 200 adults on ART for at least 6 mo at the date of status classification. Those with patient volumes below this threshold demonstrated high variability in point estimates. Facilities with systematic inconsistencies in data collection (e.g., only a single documented visit for every patient) were also excluded.

For each participating facility, we determined the best-performing definition for LTFU according to the number of days since last patient encounter. Our methodology has been described in detail elsewhere [8]. Briefly, at a set “status classification” date, all patients receiving ART at a given facility are categorized as either active or LTFU based on thresholds of 1 d to 700 d. In our previous work, we set a single status classification date (e.g., 31 December 2007) to be used across all facilities [8]. Because of the larger number of facilities incorporated into this analysis—and the great variation in their time of implementation and their dates of data export—status classification was set either at 12 mo prior to the date of a facility's last data export or 12 mo prior to the last date where data entry appeared complete.

For each threshold interval, we calculated the proportion of individuals misclassified as either “false positive” (1 – specificity) or “false negative” (1 – sensitivity) by comparing status at the classification date and status in the ensuing 12 mo. An individual who was classified as having been lost who returned to care in the ensuing 12 mo would constitute a false-positive misclassification. On the other hand, an individual who was not classified as LTFU but never returned by 12 mo would constitute a false-negative misclassification. The threshold that minimized the combined false-positive and false-negative misclassification was considered the best-performing definition for LTFU. If two or more thresholds resulted in the same misclassification, the one with shortest duration was designated as the more efficient definition.

CIs were constructed for the best-performing LTFU thresholds using a bootstrap approach. Using simple case resampling, 1,000 separate bootstrap samples were generated, and the best-performing LTFU threshold was determined for each. The percentile method was used against the resulting distributions to construct 95% CIs. In our primary analysis, we pooled data from all facilities to arrive at a standard LTFU definition. We determined the resulting differences in misclassification when this overall definition was applied at each facility.

There are currently no “gold standard” methodologies for calculating a summary LTFU measure from individual facility data. Recognizing the limitation of our pooled approach, we thus conducted two secondary analyses. For the first, facilities were weighted equally to determine a cohort summary measure, cohorts were weighted equally to determine a regional summary measure, and regions were weighted equally to arrive at an overall LTFU definition. Means were used to describe the summary LTFU definition at each step. In the second approach, we computed a weighted average of facility-specific LTFU thresholds within each IeDEA region, using the inverse of the variance of the LTFU threshold estimate from the corresponding bootstrap distribution. We then averaged these best-performing regional LTFU definitions to arrive at a summary measure. We sought to determine the robustness of our primary analysis by comparing its results to the results of these alternative approaches.

We performed stratified analyses to determine the potential impact of program characteristics on the best-performing LTFU definition. Using programmatic data gathered through the IeDEA Site Assessment Tool (10 June 2009 version)—and verification by IeDEA regional data managers and facility representatives—we described each facility according to seven key characteristics: setting (i.e., urban or rural), facility type (i.e., private or public), level of care (i.e., clinic or hospital), presence of a program to follow up missed visits (including contact tracing, telephone reminders, and/or letters), provision of free ART, availability of food supplementation, and provision of family-centered care. Facilities with partial coverage of these program services—whether for targeted populations or for only a segment of the observation period—were categorized as having those characteristics. For facilities with each characteristic, a pooled approach was used to determine the best-performing LTFU threshold and its corresponding 95% CI, the latter derived from the bootstrap sampling described previously. The relationship between optimal LTFU definition and patient volume was also described using a linear regression model.

We applied the overall LTFU definition—as described by our primary analysis—to each participating health center. The overall proportion of adults classified as LTFU was calculated for each facility, with 95% CIs determined by the exact binominal method. All analyses were performed with SAS version 9.13 (SAS Institute).

## Results

Across the six IeDEA regions, observational data were available from 510 facilities routinely providing ART to adults. Of these health care centers, 132 had sufficient patient volume to be included in our analysis (i.e., ≥200 adults on ART for at least 6 mo at time of status classification). Of these 132 facilities, 21 (16%) were excluded because of gaps in data collection and/or observed inconsistencies within the patient-level medical information.

Among the 111 facilities included in our final analysis, five were located in the Central African region (Democratic Republic of the Congo), 16 in the Eastern African region (Kenya, Tanzania, and Uganda), 72 in the Southern Africa region (Botswana, Malawi, South Africa, Zambia, and Zimbabwe), ten in the Western African region (Benin, Côte d'Ivoire, Nigeria, and Senegal), six in the Asia/Pacific region (India, Malaysia, Taiwan, and Thailand), and two in the Latin America/Caribbean region (Honduras and Mexico). Data from a total of 180,718 HIV-infected adults on ART were included. Regional characteristics—including the number of patients—are shown in Table 1, while individual facility features are available in Table S1.

**Table 1:** Characteristics of participating facilities by IeDEA region.

Characteristic	Central African Region	Eastern African Region	Southern African Region	Western African Region	Asia/Pacific Region	Latin America/Caribbean Region
**Number of countries**	1	3	5	4	4	2
**Countries represented**	Democratic Republic of the Congo	Kenya, Tanzania, Uganda	Botswana, Malawi, South Africa, Zambia, Zimbabwe	Benin, Côte d'Ivoire, Nigeria, Senegal	India, Malaysia, Taiwan, Thailand	Honduras, Mexico
**Number of facilities**	5	16	72	10	6	2
**Patients included in analysis**	3,228	21,945	132,586	20,708	1,651	600
**Setting**						
Urban	5 (100%)	15 (94%)	61 (85%)	10 (100%)	6 (100%)	2 (100%)
Rural	0 (0%)	1 (6%)	11 (15%)	0 (0%)	0 (0%)	0 (0%)
**Facility type**						
Public	0 (0%)	16 (100%)	51 (71%)	8 (80%)	5 (83%)	2 (100%)
Private	5 (100%)	0 (0%)	21 (29%)	2 (20%)	1 (17%)	0 (0%)
**Level of care**						
Clinic	5 (100%)	4 (25%)	45 (63%)	7 (70%)	0 (0%)	0 (0%)
Hospital	0 (0%)	12 (75%)	27 (38%)	3 (30%)	6 (100%)	2 (100%)
**Presence of active follow-up program**						
Yes	5 (100%)	15 (94%)	51 (71%)	8 (80%)	6 (100%)	1 (50%)
No	0 (0%)	1 (6%)	21 (29%)	2 (20%)	0 (0%)	1 (50%)
**Provision of free ART**						
Yes	5 (100%)	14 (88%)	71 (99%)	9 (90%)	2 (33%)	2 (100%)
No	0 (0%)	2 (12%)	1 (1%)	1 (10%)	4 (67%)	0 (0%)
**Food supplementation available**						
Yes	2 (40%)	13 (81%)	27 (38%)	5 (50%)	1 (17%)	1 (50%)
No	3 (60%)	3 (19%)	45 (62%)	5 (50%)	5 (83%)	1 (50%)
**Family-centered care provided**						
Yes	5 (100%)	13 (81%)	2 (3%)	7 (70%)	5 (83%)	2 (100%)
No	0 (0%)	3 (19%)	70 (97%)	3 (30%)	1 (17%)	0 (0%)

**Figure 1:**
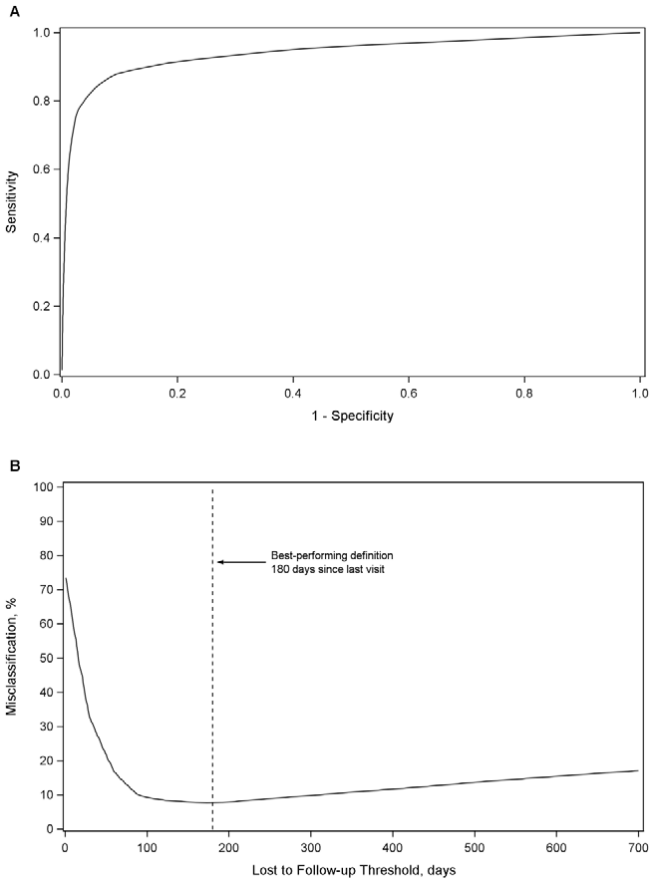
Best-performing definition for loss to follow-up. Demonstrated by receiver-operator curves (A) and misclassification (B) in the primary pooled analysis.

In our primary analysis, the best LTFU definition across all health centers was 180 d (95% CI: 173–181 d) since last visit (Figure 1A and 1B). At this threshold, sensitivity was 77.6% (95% CI: 77.3%–78.0%), specificity was 97.1% (95% CI: 97.0%–97.2%), positive predictive value was 89.9% (95% CI: 89.6%–90.2%), and negative predictive value was 93.0% (95% CI: 92.8%–93.1%). Misclassification was at its lowest at this threshold, at 7.7% (95% CI: 7.6%–7.8%). A secondary analysis that gave equal weighting to cohorts and to regions produced an optimal LTFU definition that approximated that of our primary analysis: using this approach, 175 d was found the best-performing threshold. When we weighted facilities in each region according to the inverse of the variance for each optimal LTFU definition, the best-performing threshold was 150 d.

**Figure 2:**
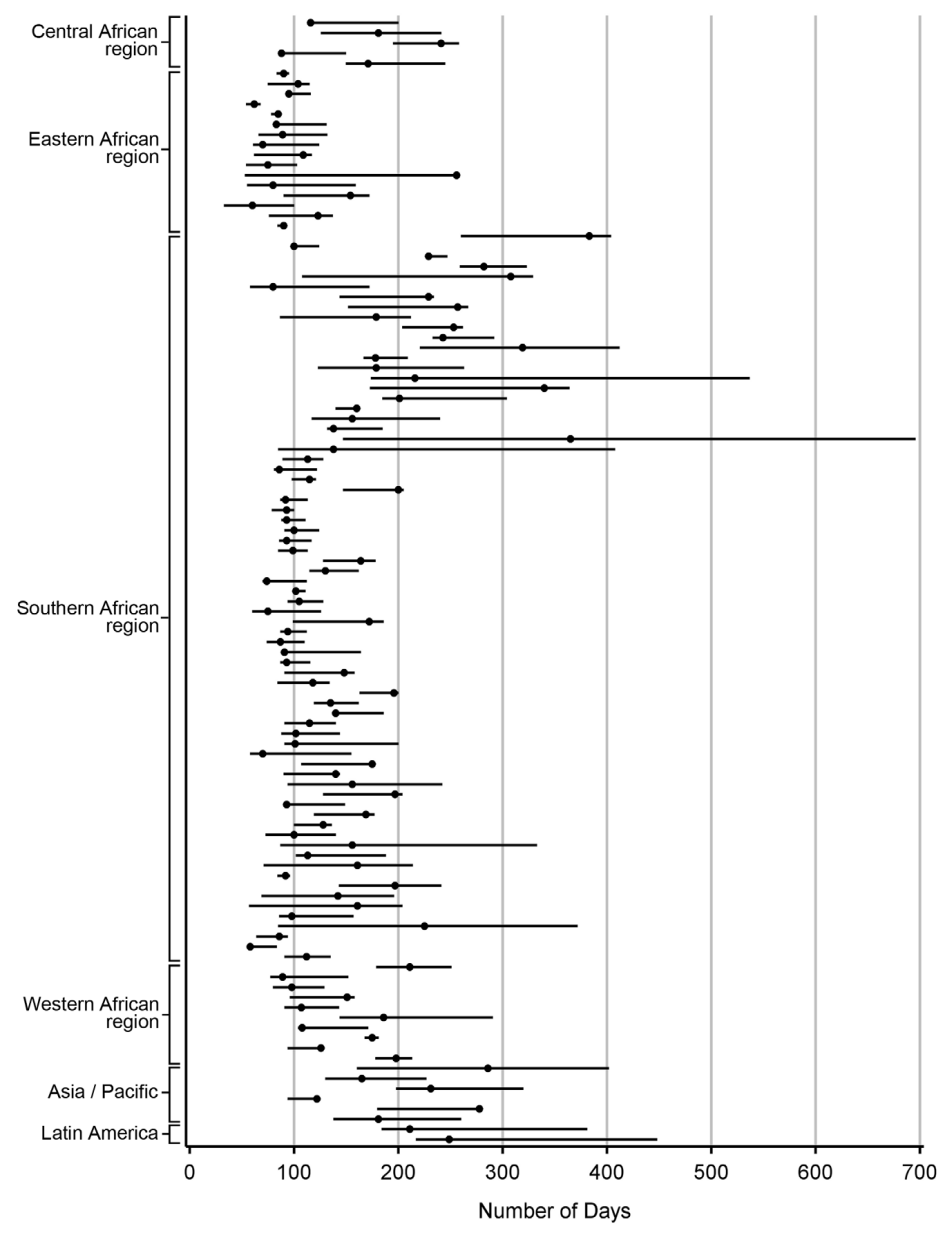
Best-performing definition of loss to follow-up by facility. Each dot represents one facility. Shown with 95% CIs determined via bootstrap modeling and grouped by IeDEA region.

**Figure 3:**
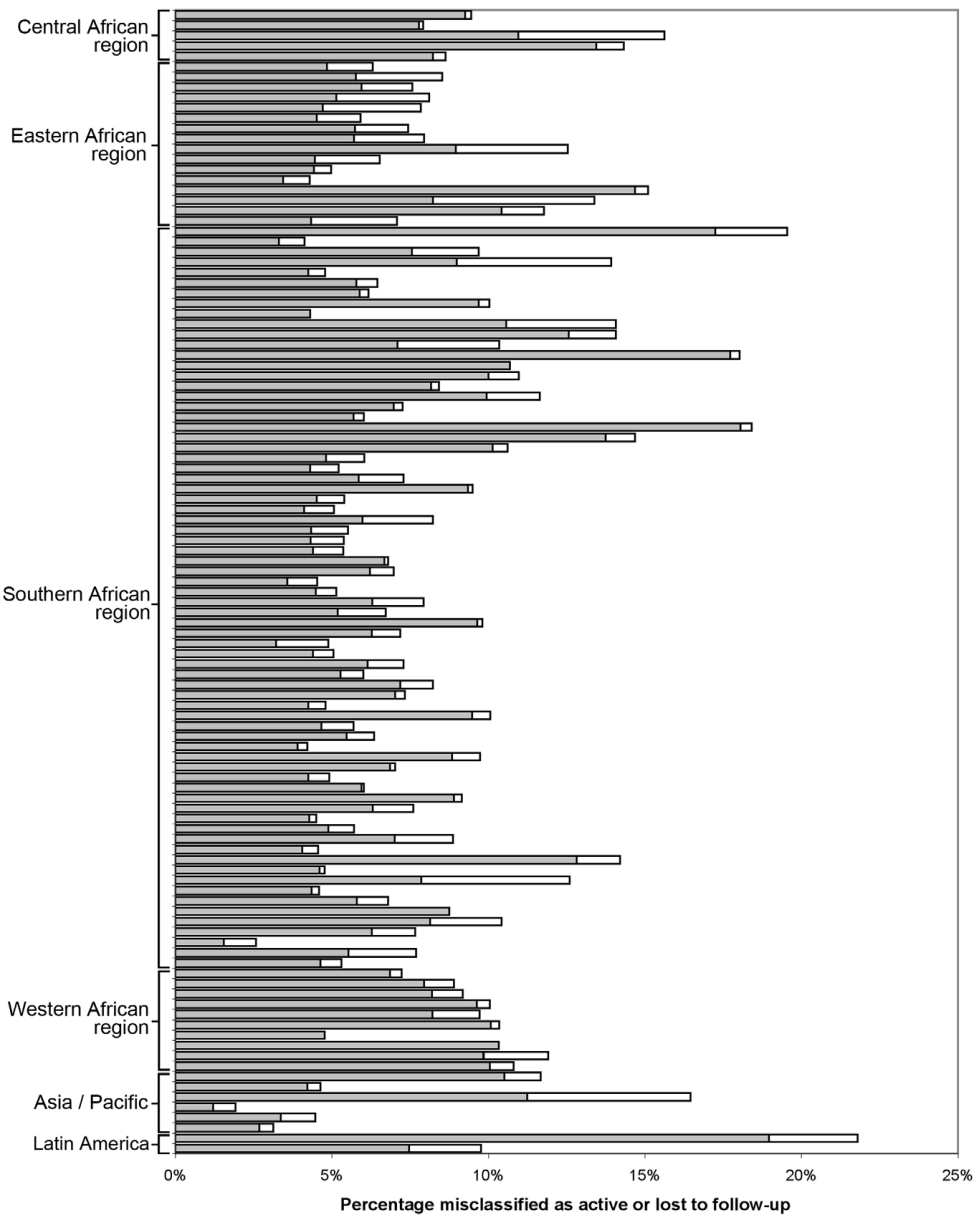
Percentage misclassified at the best-performing definition for loss to follow-up across 111 facilities, grouped by IeDEA region. The shaded portion of each bar represents the misclassification associated with each facility's best-performing LTFU definition. The white segment shows the incremental increase in misclassification when the proposed standard definition of 180 d was applied to the health center's patient population.

For each facility, we conducted analyses to empirically determine the LTFU threshold that resulted in the fewest misclassifications. Results are shown in Figure 2. The range for these facility-specific LTFU definitions ranged from 58 d to 383 d since last visit, with a mean of 150 d (95% CI: 137–163 d). The lowest misclassification at each facility ranged from 1.2% to 19.0%, with a mean of 7.2% (95% CI: 6.6%–7.8%). When the summary definition of 180 d—as calculated from our primary analysis—was applied to each facility, the observed additional increase in misclassification was between 0% and 5.2%, with a mean difference of 1.2% (95% CI: 1.0%–1.5%; Figure 3). These incremental differences in misclassification were slightly higher when the 175-d definition was applied (mean = 1.3%; 95% CI: 1.1%–1.6%) and when the 150-d definition was used (mean = 1.6%; 95% CI: 1.2%–2.1%).

**Figure 4:**
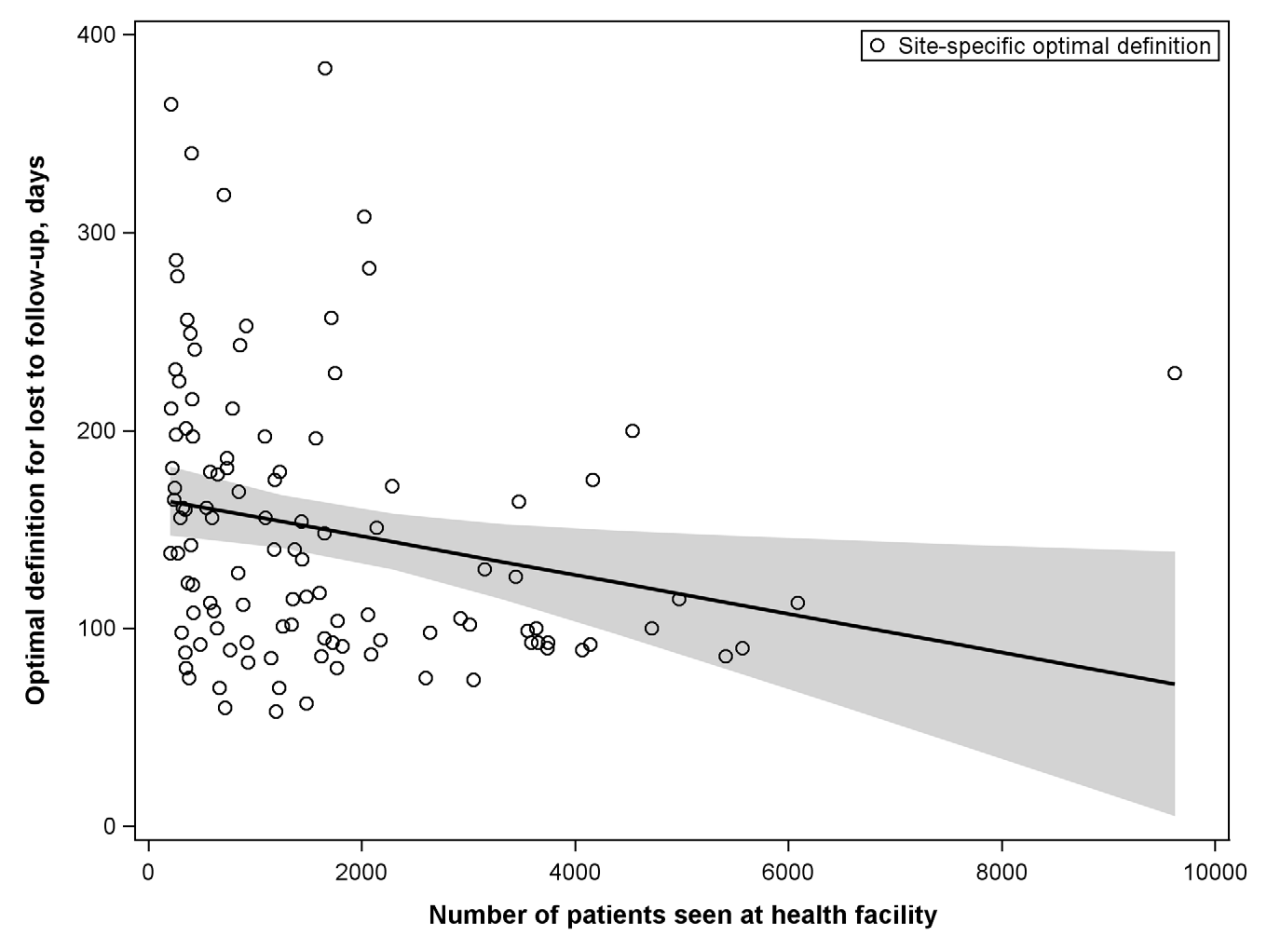
Association between patient volume and optimal definition for loss to follow-up across 111 participating facilities. The line represents the results of a linear regression model, while the shaded portion represents its 95% CI.

When we stratified according to different facility-level characteristics, only minor differences were observed in the best-performing definition for setting type or presence of an active follow-up program. Optimal LTFU definitions differed by ≥21 d when patients were categorized according to facility type (35-d difference), level of care (30-d difference), provision of free ART (21-d difference), and provision of family-centered care (31-d difference). The corresponding differences in misclassification, however, were small and not believed to be practically meaningful (Table 2). When we examined the relationship between patient volume and best-performing LTFU definitions, smaller facilities appeared to have longer optimal thresholds when compared to health centers with higher enrollments (Figure 4).

**Table 2:** Best-performing definition for loss to follow-up when patient populations are stratified according to the different characteristics of facilities at which they seek care.

Facility Characteristic	Number of Facilities	Number of Patients	Best-Performing Definition, Days	Misclassification (95% CI)
**Setting**				
Rural	12/111 (11%)	8,635	175	6.9% (6.4%–7.5%)
Urban	99/111 (89%)	172,083	180	7.7% (7.6%–7.9%)
**Facility type**				
Public	81/111 (73%)	154,618	173	7.3% (7.2%–7.5%)
Private	30/111 (27%)	26,100	208	9.6% (9.2%–9.9%)
**Level of care**				
Clinic	57/111 (51%)	84,298	150	6.8% (6.6%–6.9%)
Hospital	54/111 (49%)	96,420	180	8.6% (8.4%–8.8%)
**Presence of active follow-up program**				
Yes	86/111 (77%)	151,931	180	7.5% (7.3%–7.6%)
No	25/111 (23%)	28,787	175	8.9% (8.6%–9.3%)
**Provision of free ART**				
Yes	103/111 (93%)	174,216	180	7.6% (7.4%–7.7%)
No	8/111 (7%)	6,502	159	10.8% (10.1%–11.6%)
**Food supplementation available** a				
Yes	49/111 (44%)	100,946	180	7.0% (6.9%–7.2%)
No	62/111 (56%)	79,772	173	8.5% (8.3%–8.7%)
**Family-centered care provided**				
Yes	34/111 (32%)	37,294	150	8.9% (8.7%–9.2%)
No	77/111 (68%)	143,424	181	7.3% (7.2%–7.5%)

a Available to at least a subset of patients at the facility.

**Figure 5:**
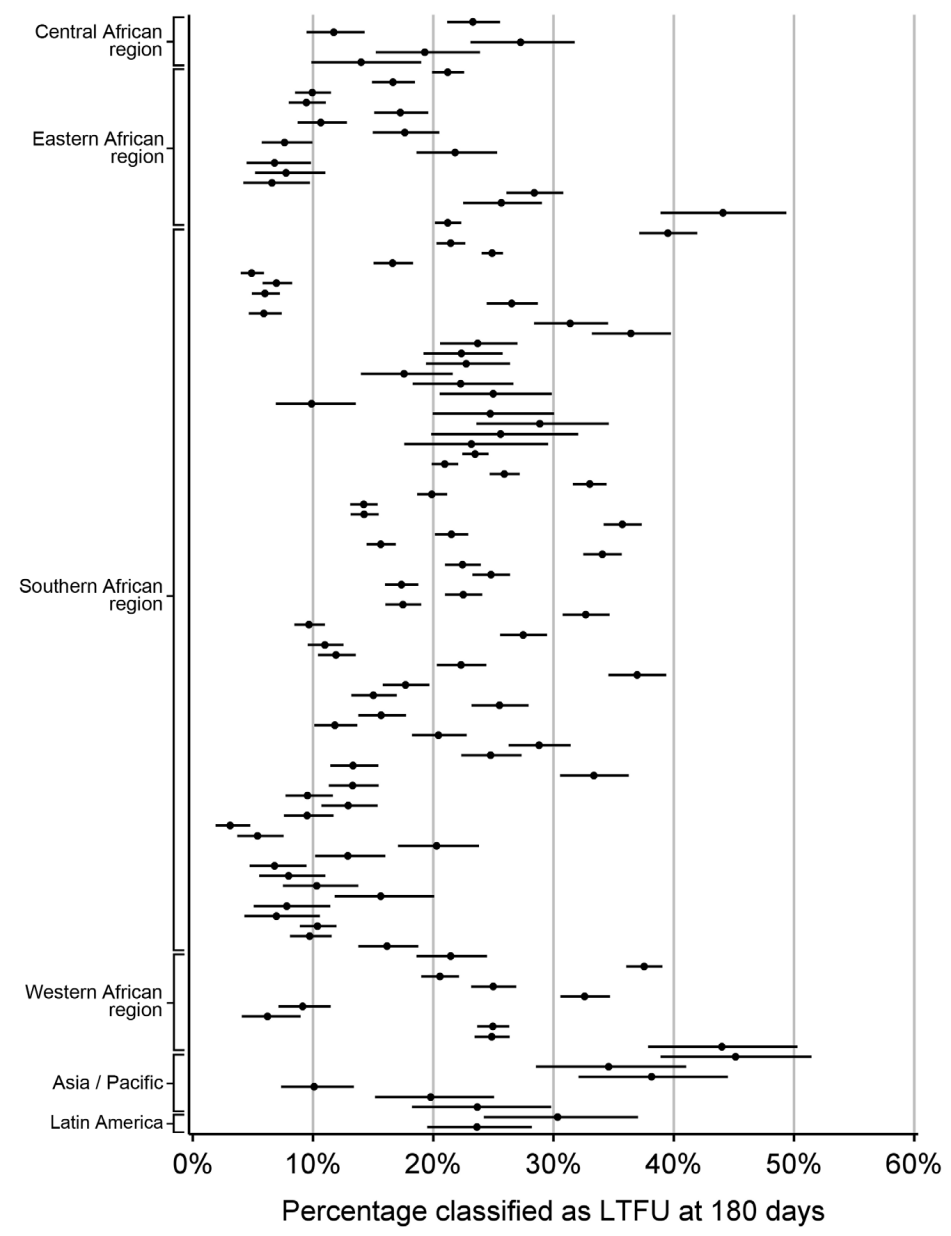
Percentage of patients classified as lost to follow-up at each of 111 participating facilities based on the proposed standard definition of 180 d since last visit, grouped by IeDEA region.

Overall, 38,615 of 180,718 (21.4%) patients were classified as LTFU at the time of status classification based on a definition of 180 d since last visit. At this threshold, the proportion of adults on ART that would be classified as LTFU ranged from 3.1% to 45.1% (mean = 19.9%; 95% CI: 18.1%–21.7%; Figure 5).

## Discussion

The objective of this analysis was to empirically determine a standard LTFU definition that could be used across ART programs worldwide. To achieve this aim, we used a methodology that minimized the inaccurate categorization of patients as either active or LTFU. In a pooled analysis of 111 facilities, a definition of 180 d for LTFU resulted in the fewest patients misclassified, a finding generally supported by our other summary approaches.

At present, there is a great deal of variability in LTFU definitions used across different settings: a standard definition for LTFU may be valuable in a number of different contexts [12]. In the area of monitoring and evaluation of ART programs, for example, managers could use a universal definition to compare program performance between facilities and/or cohorts. Such an approach would help to identify “best practices” associated with low LTFU rates, while providing the necessary framework for ongoing evaluation and quality improvement. In the area of health systems research, an empirically determined LTFU definition could provide much needed standardization to the outcome measures of clinical trials and epidemiologic studies. In contrast, a universal definition of LTFU—as proposed in this analysis—might have a more limited role for patient management. Our best-performing definition is based on the accurate categorization of individuals as active or LTFU; it is not designed to identify the optimal timing for retention activities such as patient recall or contact tracing.

We encountered methodological challenges in determining our summary LTFU definition. An analytic approach that pooled all data would take full advantage of the substantial resources available through the IeDEA Collaboration; however, larger facilities, cohorts, and/or regions might be overrepresented in the final result. An analytical approach that provided more balanced weighting across the different levels (e.g., cohorts and regions), on the other hand, would reduce the influence of the largest facilities at risk of overemphasizing the role of smaller cohorts or regions. To address this important issue, we conducted three separate analyses, each taking into account these different strengths and limitations. Two of these yielded similar results: 180 d as the best LTFU definition when all data were pooled and 175 d when cohorts and regions were given equal weighting. The third approach, which provided weighting inverse to the variance from each facility's bootstrap simulations, resulted in an optimal LTFU definition that was slightly shorter in duration (i.e., 150 d since last visit). Since large health centers exhibited the smallest variances in our analysis—and since facilities with the largest patient volumes had the shortest optimal LTFU thresholds (Figure 4)—this finding was not surprising. Because of the small difference in number of misclassifications noted between this result and that of our primary analysis (+0.2%), however, we recommend use of the ≥180-d threshold for defining LTFU.

Many ART programs have used a 6-mo absence from the health care facility to define LTFU [13]–[15], a practice supported by our analysis. Because there is no standard definition among ART programs, other thresholds have also been frequently considered. In Kenya's AMPATH (Academic Model Providing Access to Healthcare) cohort, for example, patients are categorized as LTFU if more than 3 mo have elapsed since the last clinic encounter [16],[17]. Compared to our proposed 180-d LTFU definition, such a 90-d threshold would result in only a 2.3% increase in misclassification (10.0% versus 7.7%) but a 7.3% (28.7% versus 21.4%) increase among those categorized as LTFU. If a 365-d definition for LTFU had been used—as done previously by ART-LINC, ART-CC, and IeDEA investigators [18]–[20]—misclassification would increase by 3.3% (11.0% versus 7.7%), while the proportion categorized as LTFU would decrease by 6.1% (15.3% versus 21.4%). Such differences in reported patient attrition could have an important impact on program evaluations and cohort analyses.

Our current analysis represents a substantial extension of a methodology previously applied to the large, well-characterized Lusaka ART cohort [8]. We included data from 111 health centers across three continents, increasing the external validity of our findings. We calculated the best-performing LTFU definitions for each of these facilities, demonstrating the differences that may exist even when health centers share multiple program characteristics. We also measured LTFU by the days since last visit, a metric that is more likely to be useful across programs. A LTFU definition based on “lateness” to the next scheduled clinic visit would undoubtedly have greater precision, but most electronic medical records do not routinely provide information on the next scheduled visit. Where possible, we suggest that the date of next clinical visit be included in standard program registration and reporting, particularly given its clear and important role in coordinating outreach for defaulters.

We recognize that our approach for establishing a universal definition for LTFU may overlook intricacies inherent to specific clinics and to specific patients. Appointment schedules, for example, may change over the course of treatment and may vary between health care facilities. The capacity to account for transfers between facilities may also differ, depending on the availability and sophistication of, and linkages between, electronic medical records. However, we view this “real world” perspective as a strength of our approach, particularly given the large number of clinics included in the analysis. Our final summary measure may appear imperfect for any one health center, but performance is markedly improved in the context of multiple different settings.

When our proposed universal LTFU definition (i.e., 180 d) was applied to each facility, we observed only small increases in misclassification, even when the individual health center's best-performing definition was far from 180 d. This finding can be explained by the shape of the misclassification curve (Figure 1B). When facility-specific misclassification curves were reviewed, the same general trend emerged. As the window for LTFU classification was extended, there was an initial rapid decline in misclassification, which dropped to a nadir and then gradually rose over the subsequent 200 to 300 d. This provided an extended period across which only small incremental differences are observed in misclassification.

The more accurate the categorization of active or LTFU is at the time of status classification, the shorter the optimal LTFU definition for that specific facility. When many patients returned to care after extended periods, a longer LTFU threshold was needed to minimize misclassification [8]. These trends may help to explain some of the differences observed among facilities. Characteristics thought to improve patient retention (e.g., free ART, food supplementation, and active follow-up after missed visits) were generally associated with optimal LTFU definitions that were longer (Table 2), suggesting that patients often returned to care even after a significant period had elapsed since their last clinic visit. The exception was family-centered care, where facilities that incorporated such recruitment strategies had shorter optimal LTFU definitions (150 d, versus 181 d for facilities that did not have family-centered care). Interestingly, patient volume was inversely associated with the length of the health facility's best-performing LTFU threshold. Specifically, health care centers with larger patient volumes appeared to have shorter optimal LTFU definitions. The increased waiting times typically associated with such crowded and overburdened settings likely serve as an important obstacle for retention; as a result, those on ART more quickly distinguish themselves as either active or LTFU.

We note several limitations to this analysis. First, while we advocate for establishment of a universal LTFU threshold, we recognize the marked heterogeneity in best-performing definitions among participating facilities (Figure 2). While we were reassured by the marginal differences in misclassification when the 180-d threshold was applied, it is possible that—in certain contexts—local, national, or regional definitions may be more appropriate for program evaluation. In these situations, the methodology described in this report can be used to determine specific LTFU thresholds for the populations of interest. Second, we did not include HIV-infected patients who sought care but were not yet eligible for treatment, a population that has been shown to have high rates of attrition [21],[22]. Optimal LTFU definitions for the “pre-ART” population are likely longer than for those initiating ART and should be explored further. Third, we observed instability in our point estimates when this methodology was applied to clinics with smaller volumes and/or incomplete data collection. As a result, we were unable to use data from many smaller facilities contributing data to the IeDEA Collaboration. That we were able to include the vast majority (84%) of health facilities meeting our eligibility criteria does, however, provide some confidence as to the external validity of our findings. Fourth, African facilities were heavily represented since these are the regions where program expansion has been most rapid. When the final summary definition was applied to the Asian and Latin American facilities in our study, there was a relatively low difference in misclassification (≤5%), suggesting that our findings are robust and applicable to programs outside of sub-Saharan Africa. Fifth, standardization of LTFU definitions represents only the first step in improving patient retention. Further research is needed to understand individual- and facility-level predictors of LTFU, so that at-risk populations can be identified and appropriate interventions can be evaluated [12].

A universal LTFU definition for ART program monitoring is clearly needed, but how would such standardization be best achieved? Because of the wide range of LTFU thresholds already in use [3], we advocate a top-down approach. Consensus for key monitoring and evaluation parameters (including LTFU) should first be established, based on input from program managers, policymakers, and program funders. In these deliberations, a broad range of criteria must be applied. Although we focus on the proper classification of patient status in this analysis—and believe it to be critical—other factors (e.g., clinical care implications and infrastructural demands) deserve consideration as well. Once established, buy-in from local governments and funders will be needed so that these consensus definitions are incorporated into routine program reporting. In some settings, implementation will require only minor adjustments to existing registers, electronic medical records, and data reporting systems (e.g., national-level health management information systems). The United States President's Emergency Plan for AIDS Relief, for example, already has standard reporting requirements [23] and similar measures have been adopted by local governments as well [24]. In other contexts, investment may be needed, both in terms of equipment and human resources, to ensure that such information is captured in a proper and timely fashion. Finally, such standardization will be useful only if data are routinely collected and reviewed. Ongoing monitoring is needed to ensure that feedback loops back to facilities are intact.

In conclusion, based on this large evaluation of 111 health facilities, we recommend a threshold of 180 d since the last clinic visit as a standard definition for LTFU. Harmonization of monitoring and evaluation activities in this manner is an important step towards understanding the phenomenon of patient attrition within and between cohorts worldwide. Standardization is also crucial to the development and comprehensive implementation of methodology correcting for bias in measures of program effectiveness, including assessment of mortality [25]–[27] and estimation of major disease markers such as CD4 counts. Finally, it provides the necessary framework for continued research to improve patient retention [28]–[30], so that the health gains from HIV treatment programs may be maximized and sustained.

## Supporting Information

Table S1 **Characteristics of the 111 health facilities included in this analysis.**
(PDF)

## Abbreviations

ART: antiretroviral therapy
CI: confidence interval
IeDEA: International Epidemiologic Databases to Evaluate AIDS
LTFU: lost to follow-up
