# Supplementary material for: Universal Definition of Loss to Follow-Up in HIV Treatment Programs: A Statistical Analysis of 111 Facilities in Africa, Asia, and Latin America
Source: PLoS Med. 2011 Oct 25;8(10):e1001111. doi: 10.1371/journal.pmed.1001111 (PMC3201937; doi:10.1371/journal.pmed.1001111)
Supplement: Table S1 — Characteristics of the 111 health facilities included in this analysis. (PDF) [file pmed.1001111.s001.pdf]

IeDEA Region	Health facility	Patients	Rural vs. Urban	Public vs. Private	Facility Type	Active F/U Program	Free ART provided	Food Supplementation	Family-Centered Care
**Africa, Central**	Democratic Repub Congo 1	1,481	Urban	Private	Clinic	Yes	Yes	No	Yes
	Democratic Repub Congo 2	733	Urban	Private	Clinic	Yes	Yes	Yes	Yes
	Democratic Repub Congo 3	429	Urban	Private	Clinic	Yes	Yes	Yes	Yes
	Democratic Repub Congo 4	342	Urban	Private	Clinic	Yes	Yes	No	Yes
	Democratic Repub Congo 5	243	Urban	Private	Clinic	Yes	Yes	No	Yes
**Africa, Eastern**	Kenya 1	3,740	Urban	Public	Hospital	Yes	Yes	Yes	Yes
	Kenya 2	1,772	Urban	Public	Hospital	Yes	Yes	Yes	Yes
	Kenya 3	1,649	Urban	Public	Hospital	Yes	Yes	Yes	Yes
	Kenya 4	1,480	Urban	Public	Hospital	Yes	Yes	Yes	Yes
	Kenya 5	1,147	Urban	Public	Clinic	Yes	Yes	Yes	Yes
	Kenya 6	930	Urban	Public	Hospital	Yes	No	Yes	Yes
	Kenya 7	766	Urban	Public	Clinic	Yes	Yes	Yes	Yes
	Kenya 8	667	Urban	Public	Hospital	Yes	Yes	Yes	Yes
	Kenya 9	614	Urban	Public	Clinic	Yes	Yes	Yes	Yes
	Kenya 10	382	Urban	Public	Hospital	Yes	Yes	Yes	No
	Kenya 11	361	Urban	Public	Hospital	Yes	Yes	Yes	Yes
	Kenya 12	349	Rural	Public	Clinic	Yes	Yes	Yes	Yes
	Tanzania 1	1,436	Urban	Public	Hospital	Yes	Yes	No	No
	Tanzania 2	717	Urban	Public	Hospital	Yes	No	Yes	Yes
	Tanzania 3	365	Urban	Public	Hospital	No	Yes	No	Yes
	Uganda	5,570	Urban	Public	Hospital	Yes	Yes	No	No
**Africa, Southern**	Botswana	1,652	Urban	Private	Clinic	Yes	No	No	No
	Malawi	4,720	Urban	Public	Hospital	Yes	Yes	Yes	No
	South Africa 1	9,621	Urban	Public	Hospital	Yes	Yes	Yes	No
	South Africa 2	2,068	Urban	Private	Hospital	Yes	Yes	Yes	No
	South Africa 3	2,024	Urban	Public	Clinic	Yes	Yes	Yes	No
	South Africa 4	1,765	Urban	Public	Clinic	Yes	Yes	Yes	No
	South Africa 5	1,748	Urban	Public	Clinic	Yes	Yes	No	No
	South Africa 6	1,715	Urban	Public	Hospital	Yes	Yes	Yes	No
	South Africa 7	1,232	Urban	Public	Clinic	Yes	Yes	Yes	No
	South Africa 8	917	Urban	Private	Clinic	Yes	Yes	No	No
	South Africa 9	859	Urban	Private	Hospital	No	Yes	No	No
	South Africa 10	705	Urban	Private	Clinic	Yes	Yes	No	No
	South Africa 11	649	Urban	Private	Hospital	No	Yes	No	No
	South Africa 12	580	Urban	Private	Hospital	No	Yes	No	No
	South Africa 13	410	Urban	Public	Clinic	Yes	Yes	No	No
	South Africa 14	404	Urban	Private	Clinic	Yes	Yes	No	No
	South Africa 15	352	Urban	Private	Clinic	No	Yes	No	No
	South Africa 16	344	Rural	Private	Clinic	Yes	Yes	No	No
	South Africa 17	299	Urban	Private	Clinic	Yes	Yes	No	No
	South Africa 18	277	Urban	Private	Clinic	No	Yes	No	No
	South Africa 19	211	Urban	Private	Clinic	Yes	Yes	No	No
	South Africa 20	207	Urban	Private	Clinic	Yes	Yes	No	No
	Zambia 1	6,086	Urban	Public	Clinic	Yes	Yes	Yes	No
	Zambia 2	5,412	Urban	Public	Clinic	Yes	Yes	Yes	No
	Zambia 3	4,973	Urban	Public	Hospital	Yes	Yes	No	No
	Zambia 4	4,535	Urban	Public	Hospital	Yes	Yes	No	No
	Zambia 5	4,144	Urban	Public	Clinic	Yes	Yes	Yes	No
	Zambia 6	3,743	Urban	Public	Clinic	Yes	Yes	Yes	No
	Zambia 7	3,653	Urban	Public	Clinic	Yes	Yes	Yes	No
	Zambia 8	3,637	Urban	Public	Hospital	Yes	Yes	No	No
	Zambia 9	3,590	Urban	Public	Clinic	Yes	Yes	Yes	No
	Zambia 10	3,554	Urban	Public	Clinic	Yes	Yes	Yes	No
	Zambia 11	3,473	Urban	Public	Hospital	No	Yes	No	No
	Zambia 12	3,150	Urban	Public	Hospital	Yes	Yes	No	No
	Zambia 13	3,048	Urban	Public	Clinic	Yes	Yes	Yes	No
	Zambia 14	3,009	Urban	Public	Clinic	Yes	Yes	Yes	No
	Zambia 15	2,925	Urban	Public	Hospital	Yes	Yes	No	No
	Zambia 16	2,599	Urban	Public	Clinic	Yes	Yes	Yes	No
	Zambia 17	2,282	Urban	Public	Hospital	No	Yes	No	No
	Zambia 18	2,170	Urban	Public	Clinic	Yes	Yes	Yes	No
	Zambia 19	2,085	Urban	Private	Clinic	Yes	Yes	Yes	No
	Zambia 20	1,820	Urban	Private	Clinic	Yes	Yes	No	No
	Zambia 21	1,727	Urban	Public	Clinic	Yes	Yes	Yes	No
	Zambia 22	1,649	Urban	Public	Hospital	No	Yes	No	No
	Zambia 23	1,602	Urban	Public	Hospital	No	Yes	No	No
	Zambia 24	1,566	Rural	Public	Clinic	Yes	Yes	No	No
	Zambia 25	1,439	Urban	Public	Hospital	Yes	Yes	Yes	No
	Zambia 26	1,372	Urban	Public	Hospital	No	Yes	No	No

	Zambia 27	1,352	Rural	Public	Hospital	No	Yes	No	No
	Zambia 28	1,338	Urban	Public	Clinic	Yes	Yes	No	No
	Zambia 29	1,257	Urban	Private	Clinic	Yes	Yes	Yes	No
	Zambia 30	1,222	Urban	Public	Hospital	No	Yes	No	No
	Zambia 31	1,183	Urban	Public	Hospital	No	Yes	No	No
	Zambia 32	1,177	Urban	Public	Clinic	Yes	Yes	Yes	No
	Zambia 33	1,094	Urban	Public	Hospital	No	Yes	No	No
	Zambia 34	1,091	Rural	Public	Clinic	Yes	Yes	Yes	No
	Zambia 35	921	Urban	Public	Clinic	Yes	Yes	Yes	No
	Zambia 36	844	Urban	Public	Clinic	No	Yes	No	No
	Zambia 37	841	Urban	Private	Hospital	No	Yes	No	No
	Zambia 38	642	Urban	Public	Clinic	Yes	Yes	No	No
	Zambia 39	593	Urban	Public	Clinic	Yes	Yes	Yes	No
	Zambia 40	577	Urban	Public	Hospital	No	Yes	No	No
	Zambia 41	543	Rural	Public	Clinic	Yes	Yes	No	No
	Zambia 42	484	Urban	Private	Clinic	No	Yes	No	No
	Zambia 43	414	Urban	Public	Clinic	No	Yes	No	No
	Zambia 44	397	Rural	Public	Clinic	No	Yes	No	No
	Zambia 45	320	Rural	Public	Clinic	Yes	Yes	No	No
	Zambia 46	307	Rural	Public	Hospital	No	Yes	No	No
	Zambia 47	287	Rural	Private	Clinic	Yes	Yes	No	No
	Zimbabwe 1	1,621	Urban	Private	Clinic	Yes	Yes	Yes	No
	Zimbabwe 2	1,194	Rural	Public	Hospital	Yes	Yes	No	Yes
	Zimbabwe 3	885	Rural	Public	Hospital	Yes	Yes	No	Yes
**Africa, Western**	Benin	788	Urban	Public	Hospital	Yes	Yes	Yes	No
	Cote d'Ivoire 1	4,066	Urban	Public	Hospital	Yes	Yes	No	No
	Cote d'Ivoire 2	2,638	Urban	Private	Clinic	Yes	Yes	No	Yes
	Cote d'Ivoire 3	2,140	Urban	Public	Clinic	Yes	Yes	Yes	Yes
	Cote d'Ivoire 4	2,058	Urban	Private	Hospital	No	No	Yes	Yes
	Cote d'Ivoire 5	734	Urban	Public	Clinic	Yes	Yes	No	Yes
	Cote d'Ivoire 6	419	Urban	Public	Hospital	Yes	Yes	No	Yes
	Nigeria 1	4,165	Urban	Public	Hospital	No	Yes	No	Yes
	Nigeria 2	3,441	Urban	Public	Hospital	Yes	Yes	Yes	Yes
	Senegal	259	Urban	Public	Hospital	Yes	Yes	Yes	No
**Asia**	India	257	Urban	Private	Hospital	Yes	Yes	No	Yes
	Malaysia	237	Urban	Public	Hospital	Yes	No	No	Yes
	Taiwan	249	Urban	Public	Hospital	Yes	Yes	Yes	Yes
	Thailand 1	416	Urban	Public	Hospital	Yes	No	No	No
	Thailand 2	268	Urban	Public	Hospital	Yes	No	No	Yes
	Thailand 3	224	Urban	Public	Hospital	Yes	No	No	Yes
**Latin America**	Honduras	211	Urban	Public	Hospital	Yes	Yes	Yes	Yes
	Mexico	389	Urban	Public	Hospital	No	Yes	No	Yes
